# Burden, Trends, and Future Projections of Multiple Myeloma in China from 1990 to 2023

**DOI:** 10.3390/cancers18152449

**Published:** 2026-07-30

**Authors:** Huiwen Liu, Jiazi Zhou, Hong Liu, Depei Wu

**Affiliations:** National Clinical Research Center for Hematologic Diseases, Jiangsu Key Laboratory of Hematologic Diseases, Jiangsu Institute of Hematology, The First Affiliated Hospital of Soochow University, Suzhou 215006, China; 20224232013@stu.suda.edu.cn (H.L.);

**Keywords:** multiple myeloma, global burden of disease, DALYs, epidemiological change, decomposition analysis, trends

## Abstract

Background: Multiple myeloma (MM) is a malignant plasma cell disorder associated with substantial mortality and long-term disability. With rapid population aging in China, its disease burden may be increasing, yet comprehensive long-term assessments remain limited. Methods: Using data from the Global Burden of Disease Study 2023, we evaluated trends in prevalence, incidence, deaths, and disability-adjusted life years (DALYs) of MM in China from 1990 to 2023. Age-standardized rates were analyzed using Joinpoint regression to estimate temporal trends. Results: Between 1990 and 2023, prevalence, incidence, deaths, and DALYs of MM increased markedly in China, with consistent upward trends in age-standardized rates. The burden was concentrated among older adults and was higher in males than females. Decomposition analysis showed that epidemiological changes were the primary driver of increased DALYs and deaths. Forecasting analyses suggest that although age-standardized mortality and DALY rates may gradually decline, the absolute number of deaths is expected to continue rising. Conclusions: The burden of MM in China has increased substantially over the past three decades and is projected to remain high. Strengthening early detection, risk-oriented prevention, and long-term management strategies is essential to mitigate future disease burden.

## Simple Summary

Multiple myeloma (MM) is the second most common hematological malignancy and poses an increasing public health challenge because of its chronic course and aging-related incidence. Using data from the Global Burden of Disease Study 2023 (GBD 2023), we systematically evaluated the burden of MM in China between 1990 and 2023 and projected future trends through 2045. We found that both the number of patients and age-standardized rates of prevalence, incidence, mortality, and disability-adjusted life years increased substantially over the past three decades. Older adults and men experienced the greatest disease burden. Decomposition analysis indicated that epidemiological changes, together with population aging, were major contributors to the increasing burden. Although age-standardized mortality and disability rates are projected to decline gradually, the absolute number of deaths is expected to continue rising because of population aging. These findings highlight the urgent need for earlier diagnosis, equitable access to effective therapies, and long-term disease management strategies to reduce the future burden of MM in China.

## 1. Introduction

Multiple myeloma (MM) is a malignant clonal plasma cell disorder characterized by abnormal plasma cell proliferation, monoclonal immunoglobulin secretion, and multisystem involvement, most notably osteolytic bone disease, renal dysfunction, anemia, and immune impairment [1]. Although MM accounts for only a small proportion of all malignancies, it is the second most common hematological cancer worldwide and is associated with substantial morbidity, premature mortality, and long-term healthcare utilization because of its chronic relapsing disease course [2]. Despite remarkable therapeutic advances over the past two decades, MM remains largely incurable, with repeated relapse and treatment resistance contributing to persistent disease burden and reduced quality of life.

In China, although the overall incidence of MM remains relatively low compared with many solid tumors, its absolute number of cases has increased steadily over the past three decades [3]. This increase has been driven not only by rapid population aging but also by prolonged life expectancy, urbanization, and changing demographic structures that have expanded the population at risk for age-related hematological malignancies [4]. At the same time, continuous improvements in healthcare infrastructure, nationwide medical insurance coverage, expansion of tertiary hospitals, and increasing availability of specialized hematology services have substantially enhanced diagnostic accessibility and disease management. Because MM predominantly affects older adults, with most patients diagnosed after 60 years of age, China’s rapidly aging population is expected to further increase the demand for long-term treatment, supportive care, and healthcare resources, making MM an increasingly important public health challenge. Previous studies have demonstrated considerable regional heterogeneity in MM burden across China, which may reflect differences in socioeconomic development, population age structure, healthcare accessibility, specialist availability, and the unequal adoption of novel anti-myeloma therapies [4,5]. These disparities suggest that the burden of MM is influenced not only by biological factors but also by variations in healthcare systems and resource allocation.

The diagnostic paradigm for MM has also evolved substantially during the study period. In 2003, the International Myeloma Working Group (IMWG) established standardized diagnostic criteria, and these were further revised in 2014 by incorporating the SLiM biomarkers into the traditional CRAB criteria, enabling earlier diagnosis of patients at high risk of disease progression before irreversible end-organ damage developed [6]. Simultaneously, advances in serum protein electrophoresis, immunofixation electrophoresis, serum free light chain assays, multiparameter flow cytometry, fluorescence in situ hybridization, and modern imaging modalities—including whole-body low-dose computed tomography, magnetic resonance imaging, and positron emission tomography/computed tomography—have markedly improved diagnostic sensitivity, molecular risk stratification, and disease monitoring [7]. Consequently, temporal increases in MM incidence and prevalence may reflect not only true epidemiological changes but also evolving diagnostic criteria and improved case ascertainment.

Meanwhile, the therapeutic landscape of MM has undergone a profound transformation. Since the early 2000s, the introduction of proteasome inhibitors, immunomodulatory drugs, monoclonal antibodies, autologous stem cell transplantation, maintenance therapy, and, more recently, BCMA-targeted immunotherapies has substantially prolonged progression-free and overall survival [6,8,9]. In China, successive updates to national clinical practice guidelines, inclusion of innovative anti-myeloma agents in the National Reimbursement Drug List, and improvements in supportive care have further enhanced treatment accessibility and survival outcomes [10,11]. Nevertheless, regional disparities in access to advanced therapies remain, and most patients ultimately experience disease relapse because of clonal evolution and drug resistance. As survival continues to improve, the number of individuals living with MM and experiencing long-term disease-related complications is expected to increase, further amplifying the healthcare and socioeconomic burden. Therefore, comprehensive evaluations of long-term disease burden are essential for disentangling the effects of demographic transition, evolving diagnostic standards, therapeutic advances, and healthcare policy changes. Such evidence is critical for optimizing prevention strategies, guiding healthcare resource allocation, reducing regional disparities, and supporting evidence-based cancer control policies in China.

## 2. Methods

### 2.1. Data Sources and Disease Definition

The Global Burden of Disease Study 2023 (GBD 2023) provides comprehensive and comparable estimates of disease burden worldwide from 1990 to 2023, covering 204 countries and territories, 660 subnational locations, 375 diseases and injuries, and 88 risk factors [12,13,14]. GBD 2023 applies standardized data collection, modeling, and adjustment procedures to ensure comparability across regions and time periods. Uncertainty intervals (UIs) were derived from 1000 posterior draws, with the 95% UI defined as the 2.5th and 97.5th percentiles, reflecting uncertainty arising from data sparsity, model assumptions, and parameter estimation [13].

In GBD 2023, MM was identified according to the standardized GBD cause classification and mapped to the International Classification of Diseases (ICD), corresponding to the GBD cause category encompassing ICD-10 codes C88–C90.9 [15,16]. This coding framework includes MM together with several related plasma cell and lymphoid neoplasms, such as Waldenström macroglobulinemia, smoldering multiple myeloma (SMM), mucosa-associated lymphoid tissue (MALT) lymphoma, and other closely related hematologic malignancies. The disease burden estimates analyzed in this study were obtained from the GBD 2023 cause category of “Multiple myeloma” and represented modeled population-level estimates rather than individual patient-level data. Accordingly, the present study evaluated the disease burden of the GBD-defined “Multiple myeloma” category rather than clinically confirmed MM cases alone, and separate analyses of MGUS, SMM, or other related disorders were not possible within the GBD framework.

This study focused on prevalence, incidence, mortality, DALYs, and their corresponding age-standardized rates (ASRs) for MM in China. DALYs, a composite measure of overall disease burden, were calculated as the sum of years of life lost due to premature mortality (YLLs) and years lived with disability (YLDs), expressed as: DALYs = YLLs + YLDs [17,18].

### 2.2. Joinpoint Regression Analysis

Joinpoint regression analysis was conducted to evaluate temporal trends in age-standardized mortality rates (ASMR) and age-standardized DALY rates (ASDR) for MM in China from 1990 to 2023 [19,20]. Analyses were performed using the Joinpoint Regression Program (version 5.2.0) developed by the US National Cancer Institute (NCI). This method identifies statistically significant inflection points (“joinpoints”) where trends change in magnitude or direction. The model fits a series of log-linear regression segments to annual age-standardized rates, with the optimal number of joinpoints determined using the Monte Carlo permutation test. Temporal trends within each segment were quantified by the annual percentage change (APC), whereas the overall trend during the study period was summarized by the average annual percentage change (AAPC).

### 2.3. Decomposition Analysis

The Das Gupta decomposition method was employed to quantify the relative contributions of population growth, population aging, and epidemiological changes to variations in MM mortality and DALYs in China [21]. This method decomposes the overall change in disease burden by constructing counterfactual scenarios in which population size, population age structure, and age-specific mortality or DALY rates are sequentially replaced while the remaining components are held constant. To minimize the influence of the order of replacement, the contribution of each component was estimated by averaging across all possible decomposition sequences. Population growth refers to changes attributable solely to increases in the total population size, whereas population aging reflects shifts in the population age structure toward older age groups. Epidemiological changes represent alterations in age-specific mortality and DALY rates after accounting for changes in population size and age composition. These changes encompass the combined effects of variations in disease incidence and survival, diagnostic practices, treatment effectiveness, healthcare accessibility, exposure to risk factors, and other factors influencing age-specific disease burden.

### 2.4. Forecasting Analysis

Future trends in the burden of MM were projected using the Nordpred model within an age–period–cohort (APC) regression framework [21]. This model incorporates age, period, and birth cohort effects simultaneously and has been widely applied in long-term disease burden projections. The Nordpred model requires population data stratified by age and calendar year to estimate future disease burden. In this study, population denominators and projected population data were obtained from the GBD population projections for China, which provide annual population estimates and forecasts by sex and 5-year age groups. These projected demographic data were used as the reference population for forecasting future numbers of deaths and DALYs attributable to MM. Based on historical data, we projected the number of deaths and DALYs attributable to MM in China over the next two decades to provide evidence for future prevention strategies and healthcare resource planning. The Nordpred R package was used to generate forecasts for deaths and DALYs over the next two decades. All data cleaning, statistical analyses, and figure generation were performed using R software (version 4.2.1).

## 3. Results

### 3.1. Burden of MM in China, 1990–2023

Over the past 34 years, the prevalence of MM in China increased from 6059.14 cases (95% UI: 3081.37–12,310.51) in 1990 to 84,707.26 cases (95% UI: 40,874.38–145,818.09) in 2023, representing a 1298% increase. The percentage increase was slightly higher among males (1321%) than females (1268%). During the same period, incident cases increased by 692%, with a 683% increase in males and a 705% increase in females.

Total DALYs attributable to MM rose from 70,649.54 (95% UI: 46,072.50–124,986.98) in 1990 to 319,996.92 (95% UI: 236,765.38–384,134.55) in 2023, corresponding to a 353% increase. DALYs increased by 347% in males and 362% in females. Deaths from MM increased by 437% over the study period, with increases of 417% among males and 465% among females.

The ASDR increased from 7.18 per 100,000 population (95% UI: 4.69–12.74) in 1990 to 14.30 per 100,000 (95% UI: 10.68–17.19) in 2023, with an estimated annual percent change (EAPC) of 1.38 (95% CI: 0.84–1.92). The EAPCs for age-standardized prevalence rate (ASPR), age-standardized incidence rate (ASIR), and ASMR were 5.19 (95% CI: 4.73–5.64), 2.97 (95% CI: 2.48–3.46), and 1.52 (95% CI: 0.98–2.06), respectively (Table 1).

### 3.2. Age and Sex Distribution of MM in China, 2023

**Figure 1:**
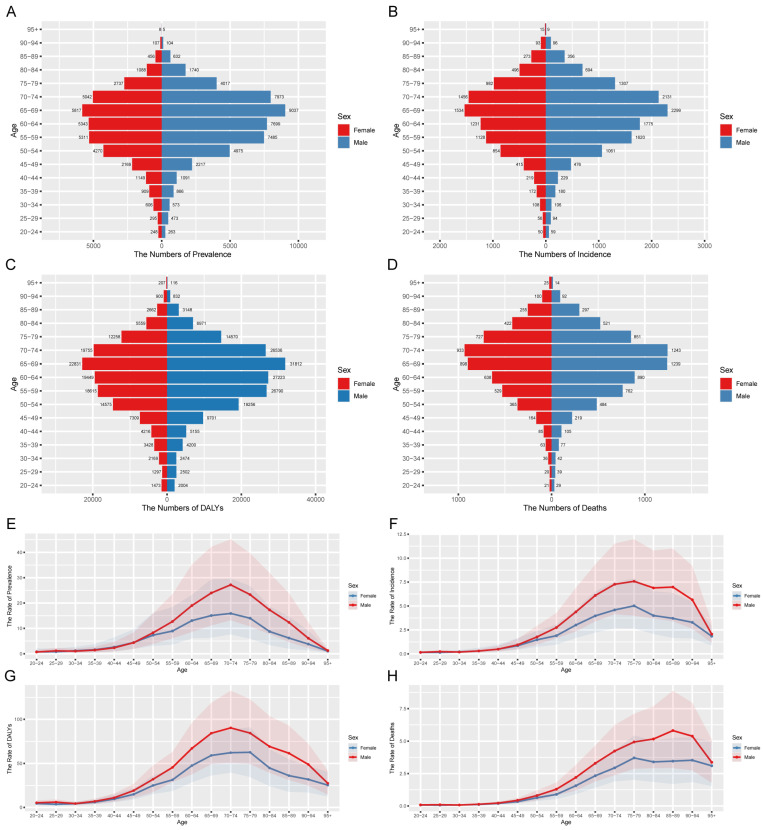
Age-specific burden of multiple myeloma in China in 2023. (**A**) Number of prevalent cases by age group and sex. (**B**) Number of incident cases by age group and sex. (**C**) Number of disability-adjusted life years (DALYs) by age group and sex. (**D**) Number of deaths by age group and sex. (**E**) Age-specific prevalence rate per 100,000 population. (**F**) Age-specific incidence rate per 100,000 population. (**G**) Age-specific DALY rate per 100,000 population. (**H**) Age-specific mortality rate per 100,000 population.

In 2023, prevalence, incidence, and DALYs of MM peaked in the 65–69 age group, reaching 9037, 2299, and 31,812 cases in males and 5817, 1534, and 22,831 cases in females, respectively. Deaths peaked in the 70–74 age group, with 1243 deaths among males and 933 among females (Figure 1A–D).

The 70–74 age group exhibited the highest prevalence rate and DALYs rate, whereas the highest incidence rate was observed in the 75–79 age group, and the highest mortality rate occurred in the 85–89 age group. Among individuals aged 50 years and older, males consistently exhibited higher prevalence rate, incidence rate, and mortality rate than females. In addition, DALYs rate was significantly higher in males than in females among those aged 45 years and above (Figure 1E–H).

### 3.3. Temporal Trends in MM in China, 1990–2023

**Figure 2:**
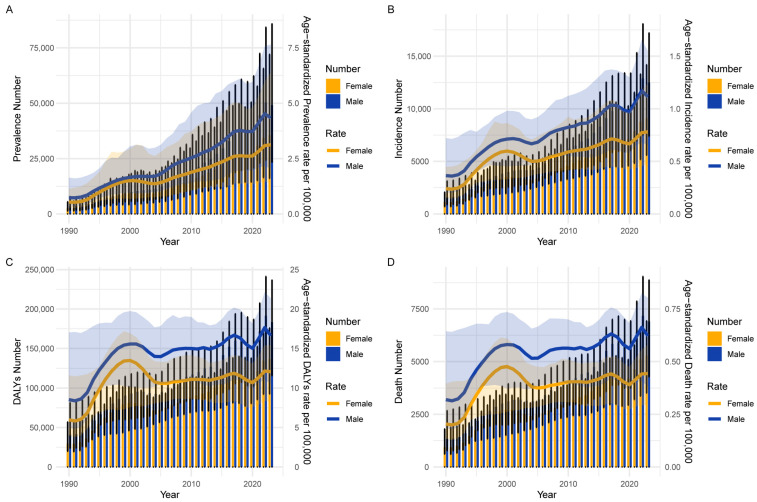
Temporal trends in the numbers and age-standardized rates of prevalence, incidence, DALYs, and deaths due to multiple myeloma in China from 1990 to 2023. (**A**) Number of prevalent cases and age-standardized prevalence rate (ASPR) by sex over time. (**B**) Number of incident cases and age-standardized incidence rate (ASIR) by sex over time. (**C**) Number of DALYs and age-standardized DALY rate (ASDR) by sex over time. (**D**) Number of deaths and age-standardized mortality rate (ASMR) by sex over time.

From 1990 to 2023, prevalence, incidence, DALYs, and deaths from MM increased rapidly in both sexes. Notably, incidence, DALYs, and deaths exhibited pronounced growth peaks between 1995 and 2000. Throughout the study period, males consistently experienced higher absolute burdens than females. Age-standardized rates demonstrated trends broadly consistent with those observed for absolute numbers (Figure 2A–D).

### 3.4. Age-Specific Trends in MM in China, 1990–2023

**Figure 3:**
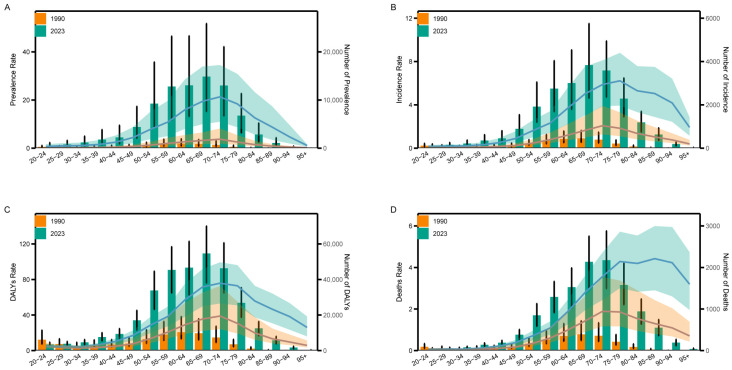
Age-specific comparisons of numbers and age-specific rates of prevalence, incidence, DALYs, and deaths due to multiple myeloma in China in 1990 and 2023. (**A**) Age-specific rates and numbers of prevalence. (**B**) Age-specific rates and numbers of incidence. (**C**) Age-specific rates and numbers of DALYs. (**D**) Age-specific rates and numbers of deaths.

Compared with 1990, age-specific prevalence, incidence, DALY, and mortality rates were higher in 2023 across most adult age groups. Nevertheless, the burden remained strongly age-dependent and increased markedly after middle age, with the majority of the burden concentrated among older adults. In 2023, the prevalence and DALY rates peaked at 70–74 years, the incidence rate peaked at 75–79 years, and the mortality rate peaked at 85–89 years. Thus, the temporal increase was broadly distributed across age groups but did not indicate an absence of age specificity (Figure 3A–D).

### 3.5. Joinpoint Regression Analysis of MM Trends in China, 1990–2023

**Figure 4:**
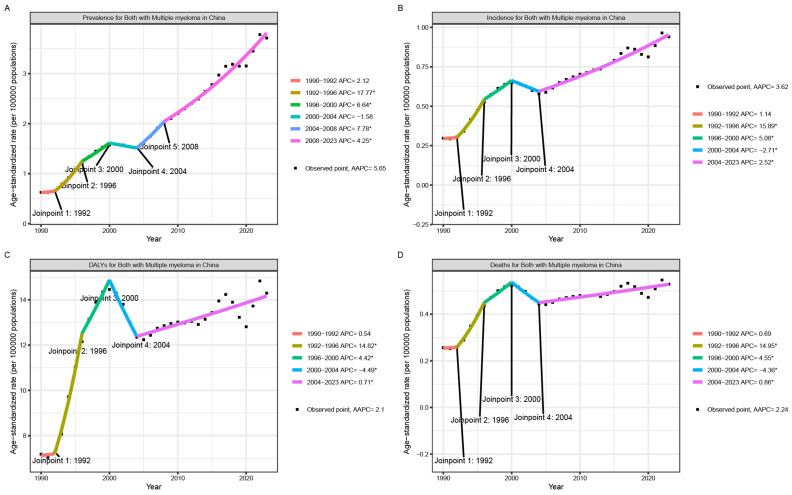
Joinpoint regression analysis of age-standardized rates of prevalence, incidence, DALYs, and deaths due to multiple myeloma in China from 1990 to 2023. (**A**) ASPR. (**B**) ASIR. (**C**) ASDR. (**D**) ASMR. * indicates statistical significance (*p* < 0.05).

Joinpoint regression analysis revealed significant upward trends in ASPR, ASIR, ASDR, and ASMR for MM in China from 1990 to 2023, with the average annual percent change (AAPC) of 5.65, 3.62, 2.10, and 2.24, respectively (Figure 4A–D). The most rapid increases occurred between 1992 and 1996, during which annual percent change (APC) for ASPR, ASIR, ASDR, and ASMR reached 17.77, 15.89, 14.82, and 14.95, respectively.

### 3.6. Decomposition of Changes in MM Deaths and DALYs in China, 1990–2023

**Figure 5:**
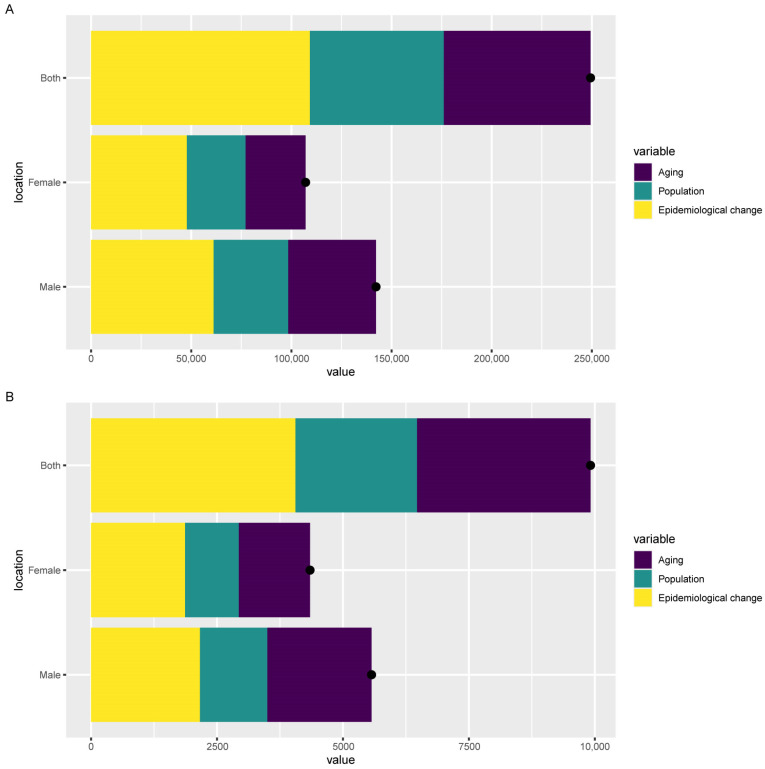
Decomposition of changes in the number of DALYs (**A**) and deaths (**B**) due to multiple myeloma in China by sex from 1990 to 2023.

Decomposition analysis indicated that epidemiological changes were the dominant contributors to increases in MM DALYs and deaths over the past 34 years, followed by population aging and population growth (Figure 5A,B). For DALYs, epidemiological changes contributed 109,195.45 (43.79%), aging contributed 73,293.51 (29.39%), and population growth contributed 66,858.42 (26.81%), resulting in a total increase of 249,347.38 DALYs, including 142,252.63 among males and 107,094.75 among females. For deaths, epidemiological changes accounted for 4055.62 (40.90%), aging for 3445.32 (34.75%), and population growth for 2414.04 (24.35%), leading to a total increase of 9914.99 deaths, with 5568.13 among males and 4346.85 among females.

### 3.7. Prediction Analysis of MM Burden in China, 2024–2045

**Figure 6:**
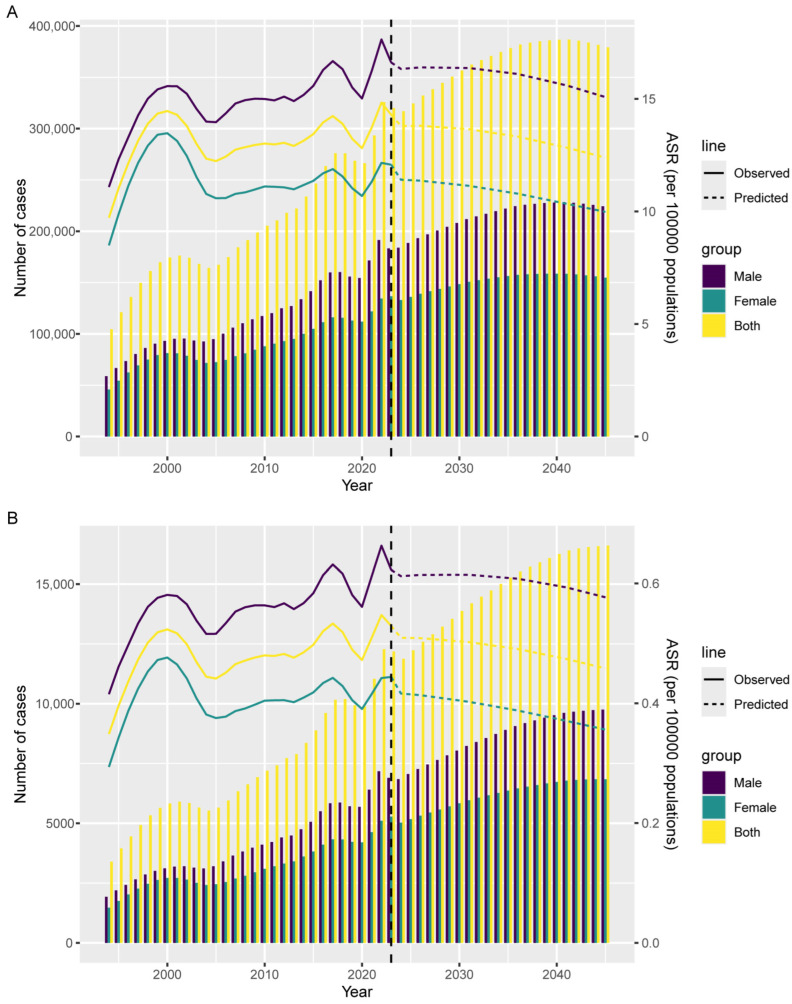
Projected trends in the burden of multiple myeloma in China through 2045. (**A**) Projected number of DALYs and ASDR by year and sex. (**B**) Projected number of deaths and ASMR by year and sex.

Forecasting analyses suggested that DALYs attributable to MM in China will initially increase and subsequently decline between 2024 and 2045, whereas deaths are projected to increase steadily over time. In contrast, ASDR and ASMR are expected to decline gradually (Figure 6A,B). By 2045, DALYs among males are projected to reach 224,338, with an ASDR of 15.08 per 100,000 population, while DALYs among females are projected to reach 154,777, with an ASDR of 9.98 per 100,000. Projected deaths are 9755 among males (ASMR: 0.58 per 100,000) and 6841 among females (ASMR: 0.36 per 100,000).

## 4. Discussion

From 1990 to 2023, the burden of MM in China increased substantially, with marked rises in prevalence, incidence, deaths, and DALYs, accompanied by consistently positive EAPCs across all age-standardized rates. Unlike many malignancies in which age-standardized rates decline despite increasing case numbers due to demographic aging, MM in China showed concurrent growth in both absolute burden and population-adjusted risk [4,16]. This pattern suggests that the expansion of disease burden may not be explained by demographic change alone but may also reflect broader shifts in epidemiological and diagnostic contexts. Joinpoint regression further confirmed persistent upward trends in ASPR, ASIR, ASDR, and ASMR over the past three decades, with the most pronounced increases occurring during the early 1990s. This early acceleration may partly reflect improvements in diagnostic capacity, including wider availability of serum protein electrophoresis, immunofixation, and bone marrow examinations, which enhanced the detection and reporting of plasma cell disorders [5,15,22,23]. Collectively, these findings indicate that MM is emerging as an increasingly important hematologic malignancy in China, posing growing challenges for long-term disease management and healthcare planning.

Compared with previous GBD-based studies of MM in China, the present study provides several important advances beyond simply updating the data source [4,24]. By incorporating GBD 2023 estimates and extending the observation period through 2023, we provide a more up-to-date assessment of the evolving disease burden. More importantly, rather than describing temporal trends alone, we quantitatively disentangled the relative contributions of population growth, population aging, and epidemiological changes using Das Gupta decomposition analysis, thereby clarifying the major drivers underlying the increasing burden. Furthermore, by integrating Joinpoint regression, decomposition analysis, and Nordpred forecasting within a unified analytical framework, this study links historical trend identification with interpretation of driving forces and future burden prediction. This integrated analytical framework extends beyond descriptive epidemiology by identifying the determinants of historical disease burden and projecting future healthcare demand, thereby providing a stronger evidence base for precision public health planning and healthcare resource allocation in China.

Age- and sex-specific analyses revealed that the burden of MM is heavily concentrated among older adults. Prevalence, incidence, and DALYs peaked at ages 65–69 years, whereas deaths peaked slightly later at ages 70–74 years, reflecting the typical clinical course in which disease progression and treatment resistance gradually lead to mortality over time. Age-standardized indicators further demonstrated a progressive increase in disease severity with advancing age, with the highest ASIR observed in individuals aged 75–79 years and the highest ASMR among those aged 85–89 years. Across nearly all adult age groups, men consistently exhibited higher ASPR, ASIR, ASDR, and ASMR than women, indicating a stable male predominance in both disease occurrence and health loss [25]. This sex disparity has been reported in several epidemiological studies and may be associated with differences in occupational exposures, lifestyle-related risk factors, immune regulation, and hormonal influences [2]. In addition, men generally present with a higher prevalence of smoking, alcohol consumption, metabolic disorders, and cardiovascular comorbidities, all of which may adversely affect treatment tolerance and survival [26]. Delayed healthcare-seeking behavior and lower utilization of preventive medical services among men may also contribute to later-stage diagnosis and poorer prognosis, thereby resulting in persistently higher mortality and DALY burdens across most adult age groups [27,28,29].

Decomposition analysis provided further insight into the drivers of the increasing disease burden. Epidemiological change contributed the largest share of the rise in DALYs and deaths, exceeding the effects of population aging and population growth. This finding suggests that shifts in underlying risk patterns, diagnostic practices, or disease survival may have played an important role in shaping the observed trends [2]. Several factors may potentially contribute to these changes, including improvements in disease recognition, increasing prevalence of metabolic disorders such as obesity and diabetes, environmental exposures, and changes in population longevity that extend the period at risk for plasma cell malignancies [4]. Forecasting analyses indicate that although ASDR and ASMR may gradually decline in the coming decades, the absolute number of deaths is projected to continue increasing, particularly among men. This projected divergence between standardized rates and absolute burden reflects the continuing impact of population aging and highlights the likelihood that healthcare systems will face sustained demand for diagnosis, treatment, and long-term care of patients with MM [30].

These findings carry important implications for public health policy and clinical management. As China continues to experience rapid demographic aging, the healthcare burden associated with MM is likely to expand further. Strengthening early recognition of plasma cell disorders, improving access to hematologic diagnostic services, and optimizing treatment strategies for older patients will therefore be essential to mitigate the future disease burden [4]. In addition, expanding access to novel therapeutic agents and improving long-term survivorship management may contribute to reducing disability and mortality associated with the disease [31]. In recent years, China has implemented several initiatives that may help alleviate the burden of MM. The continuous expansion of the National Reimbursement Drug List has substantially improved patient access to novel anti-myeloma therapies, including proteasome inhibitors, immunomodulatory drugs, and anti-CD38 monoclonal antibodies [3,4,32,33]. Meanwhile, the establishment of regional hematologic disease centers, standardized diagnosis and treatment programs, multidisciplinary care models, and the publication of updated national clinical guidelines have promoted earlier diagnosis and more standardized management across healthcare settings. Continued optimization of these strategies, together with strengthened health education and surveillance among older high-risk populations, may further improve long-term outcomes and reduce the future burden of MM in China.

Several limitations of this study should be acknowledged. First, the disease burden estimates were derived from the GBD 2023 cause category of “Multiple myeloma,” which is mapped to ICD-10 codes C88–C90.9 rather than the diagnostic definition of clinically confirmed MM alone. Consequently, this category may also include other plasma cell and lymphoid disorders, such as Waldenström macroglobulinemia, SMM, MALT lymphoma, and other related hematologic malignancies. Because the GBD database provides only aggregated estimates for this broad cause category, the burden attributable exclusively to clinically diagnosed MM could not be distinguished, representing an inherent limitation of the GBD framework. Second, the estimates were derived from the GBD 2023 modeling framework and are therefore subject to uncertainties related to data availability, potential underreporting, and model assumptions, particularly in earlier years and older age groups. Third, the current GBD framework provides limited attribution of specific risk factors for MM, restricting further exploration of environmental, metabolic, or occupational determinants of disease risk. Fourth, forecasting analyses are based on historical trends and assume relative stability in underlying patterns, and therefore may not fully account for future advances in therapeutic strategies or changes in healthcare policy that could substantially alter disease outcomes.

## 5. Conclusions

Based on a comprehensive analysis of GBD 2023 data, the burden of MM in China increased substantially between 1990 and 2023, as evidenced by sustained rises in prevalence, incidence, deaths, and DALYs, accompanied by significant upward trends in all ASRs (ASPR, ASIR, ASDR, and ASMR). This concurrent increase in both absolute numbers and ASRs indicates that the growing burden of MM in China cannot be attributed solely to population aging, but rather reflects a genuine increase in disease risk. The burden was disproportionately concentrated among older adults, particularly those aged 65 years and above, with males consistently experiencing higher mortality and DALYs than females across most age groups. Decomposition analysis identified epidemiological changes as the dominant driver of increasing DALYs and deaths, surpassing the contributions of population aging and growth. Forecasts suggest that although ASDR and ASMR may gradually decline, the absolute number of deaths will continue to rise, especially among males. Overall, MM is transitioning from a relatively rare hematologic malignancy to a high-burden chronic cancer in China, underscoring the urgent need for strengthened early diagnosis, risk-targeted prevention, and long-term standardized management, particularly for older and male populations.

## Figures and Tables

**Table 1:** All-age numbers and age-standardized rates of prevalence, incidence, mortality, and disability-adjusted life years (DALYs) for multiple myeloma in China, 1990–2023.

Indicator	Sex	Cases in 1990 (95% UI)	Cases in 2023 (95% UI)	Percentage Change	ASR per 100,000 in 1990 (95% UI)	ASR per 100,000 in 2023 (95% UI)	EAPC (95% CI)
Prevalence	Both	6059.14 (3081.37–12,310.51)	84,707.26 (40,874.38–145,818.09)	12.98	0.63 (0.32–1.27)	3.71 (1.78–6.5)	5.19 (4.73–5.64)
	Female	2599.8 (1371.57–5492.19)	35,555.8 (16,066.77–72,069.8)	12.68	0.53 (0.28–1.13)	3.13 (1.39–6.39)	4.84 (4.26–5.42)
	Male	3459.34 (1678.99–7791.12)	49,151.46 (23,388.14–85,850.67)	13.21	0.73 (0.35–1.64)	4.34 (2.04–7.65)	5.44 (5.08–5.79)
Incidence	Both	2723.19 (1670.98–4730.87)	21,574.43 (14,296.28–30,466.87)	6.92	0.3 (0.18–0.52)	0.94 (0.62–1.34)	2.97 (2.48–3.46)
	Female	1128.29 (704.44–2073.48)	9082.68 (5582–14,185.97)	7.05	0.24 (0.15–0.45)	0.78 (0.48–1.24)	2.77 (2.16–3.39)
	Male	1594.9 (894.33–3131.02)	12,491.75 (7399.5–17,209.96)	6.83	0.36 (0.2–0.72)	1.11 (0.66–1.55)	3.07 (2.68–3.45)
DALYs	Both	70,649.54 (46,072.5–124,986.98)	319,996.92 (236,765.38–384,134.55)	3.53	7.18 (4.69–12.74)	14.3 (10.68–17.19)	1.38 (0.84–1.92)
	Female	29,610.71 (19,919.64–56,975.27)	136,705.46 (92,562.13–175,903.93)	3.62	5.97 (3.96–11.6)	12.07 (8.21–15.4)	1.17 (0.52–1.83)
	Male	41,038.83 (23,942.54–81,488.65)	183,291.46 (115,840.39–236,718.18)	3.47	8.53 (4.98–17.03)	16.62 (10.52–21.35)	1.51 (1.07–1.96)
Death	Both	2268.82 (1479.33–4021.09)	12,183.8 (9075.6–14,757.62)	4.37	0.26 (0.17–0.46)	0.53 (0.39–0.64)	1.52 (0.98–2.06)
	Female	934.66 (614.85–1804.25)	5281.52 (3513.41–6930.01)	4.65	0.2 (0.13–0.4)	0.44 (0.3–0.58)	1.43 (0.76–2.09)
	Male	1334.15 (776.45–2676.91)	6902.29 (4285.17–8874.4)	4.17	0.32 (0.19–0.64)	0.62 (0.38–0.8)	1.53 (1.09–1.97)

## Data Availability

The datasets presented in this study are available online at http://ghdx.healthdata.org/gbd-results-tool (accessed on 1 March 2026). Further information can be directed to the corresponding author.
